# Fully automated computational measurement of noise in positron emission tomography

**DOI:** 10.1007/s00330-023-10056-w

**Published:** 2023-08-30

**Authors:** Thomas Sartoretti, Stephan Skawran, Antonio G. Gennari, Alexander Maurer, André Euler, Valerie Treyer, Elisabeth Sartoretti, Stephan Waelti, Moritz Schwyzer, Gustav K. von Schulthess, Irene A. Burger, Martin W. Huellner, Michael Messerli

**Affiliations:** 1 Department of Nuclear Medicine, University Hospital Zurich, Rämistrasse 100, CH-8091 Zurich, Switzerland; 2 University of Zurich, Zurich, Switzerland; 3 Institute of Diagnostic and Interventional Radiology, University Hospital Zurich, Zurich, Switzerland; 4 Department of Radiology and Nuclear Medicine, Children’s Hospital of Eastern Switzerland, St. Gallen, Switzerland; 5 Health Sciences and Technology, Institute of Food, Nutrition and Health, ETH Zurich, Zurich, Switzerland; 6 Department of Nuclear Medicine, Kantonsspital Baden, Baden, Switzerland

**Keywords:** Positron emission tomography, Noise, Algorithms, Dose reduction, Image enhancement

## Abstract

**Objectives:**

To introduce an automated computational algorithm that estimates the global noise level across the whole imaging volume of PET datasets.

**Methods:**

[^18^F]FDG PET images of 38 patients were reconstructed with simulated decreasing acquisition times (15–120 s) resulting in increasing noise levels, and with block sequential regularized expectation maximization with beta values of 450 and 600 (Q.Clear 450 and 600). One reader performed manual volume-of-interest (VOI) based noise measurements in liver and lung parenchyma and two readers graded subjective image quality as sufficient or insufficient. An automated computational noise measurement algorithm was developed and deployed on the whole imaging volume of each reconstruction, delivering a single value representing the global image noise (Global Noise Index, GNI). Manual noise measurement values and subjective image quality gradings were compared with the GNI.

**Results:**

Irrespective of the absolute noise values, there was no significant difference between the GNI and manual liver measurements in terms of the distribution of noise values (*p* = 0.84 for Q.Clear 450, and *p* = 0.51 for Q.Clear 600). The GNI showed a fair to moderately strong correlation with manual noise measurements in liver parenchyma (*r* = 0.6 in Q.Clear 450, *r* = 0.54 in Q.Clear 600, all *p* < 0.001), and a fair correlation with manual noise measurements in lung parenchyma (*r* = 0.52 in Q.Clear 450, *r* = 0.33 in Q.Clear 600, all *p* < 0.001). Classification performance of the GNI for subjective image quality was AUC 0.898 for Q.Clear 450 and 0.919 for Q.Clear 600.

**Conclusion:**

An algorithm provides an accurate and meaningful estimation of the global noise level encountered in clinical PET imaging datasets.

**Clinical relevance statement:**

An automated computational approach that measures the global noise level of PET imaging datasets may facilitate quality standardization and benchmarking of clinical PET imaging within and across institutions.

**Key Points:**

• *Noise is an important quantitative marker that strongly impacts image quality of PET images.*

• *An automated computational noise measurement algorithm provides an accurate and meaningful estimation of the global noise level encountered in clinical PET imaging datasets.*

• *An automated computational approach that measures the global noise level of PET imaging datasets may facilitate quality standardization and benchmarking as well as protocol harmonization.*

## Introduction

Positron emission tomography (PET) is an established diagnostic imaging modality for the assessment of a wide array of oncological and inflammatory diseases [1]. Image quality may considerably impact the diagnostic accuracy and evaluability of PET images. PET image quality and perception are influenced by various factors such as spatial resolution, contrast, signal-to-noise ratio (SNR), radiotracer uptake, motion artifacts, reconstruction algorithm, and scanner design, calibration, and performance. These factors are interdependent and optimizing one may require making compromises with the others to obtain high-quality PET images [2]. Noise, which is the random variation of the signal intensity, is an important baseline parameter that can affect PET image quality [3, 4]. Noise, among other factors, is influenced by acquisition time, administered activity [5], and reconstruction methods [6]. High noise levels can reduce the SNR and image contrast, making it more difficult to detect and interpret the radiotracer uptake. Therefore, minimizing noise in PET imaging is crucial to obtain clear and accurate images [3]. Thus, in clinical routine and more often in research, image noise of PET images is measured to objectivize image quality [7, 8]. This can be achieved by means of regional, slice-based manual measurements (i.e., by placing volumes-of-interest (VOI)). While such measurements are an established and simple means of quantifying image noise [7–11], they are reader-dependent and thus also tedious and time-consuming. In addition, only a small part of the imaging volume is considered. In this regard, a fully automated computational approach enabling an accurate estimation of the image noise level across the whole imaging volume would be highly desirable. This would allow the user to estimate the global image noise level of PET datasets effortlessly in a high-throughput fashion and could even be envisioned as a fully integrated tool in PET/CT systems to monitor and adjust acquisition protocols for a stable optimized image quality.

The objective of our study was to develop an algorithm that enables the automated computational estimation of the noise level across the whole imaging volume of PET datasets. Furthermore, we sought to assess the performance of this method by correlating it with manual noise measurements in liver and lung parenchyma and by comparing it with image quality as determined subjectively by expert readers.

## Materials and methods

### Study population

Thirty-eight patients who underwent clinically indicated [^18^F]FDG PET/CT imaging between March and April 2021 were retrospectively selected. There were no specific inclusion criteria except the full availability of clinical and imaging data. The patients included in the study were part of an earlier investigation by our institution evaluating an unrelated image quality classifier (currently under review). Written informed consent for the scientific use of medical data was obtained from all patients. The study was approved by the local ethics committee (BASEC 2021–00444, Cantonal Ethics Committee Zürich, Switzerland).

### PET acquisition and reconstruction

Examinations were performed on a latest generation six-ring digital detector PET/CT scanner (Discovery MI Gen 2, GE Healthcare). A body mass index (BMI)-adapted 18F-FDG dosage protocol was used as outlined in detail elsewhere [6]. To generate standardized uptake value (SUV) images with increasing noise levels, five datasets were reconstructed from each exam by unlisting list mode data, resulting in reduced emission counts equivalent to 120 s, 90 s, 60 s, 30 s, and 15 s acquisition time per bed position. For each patient, 6–8 bed positions were acquired (depending on patient size), with an overlap of 23% (17 slices) [6]. Furthermore, images were reconstructed with a proprietary reconstruction kernel using block sequential regularized expectation maximization (Q.Clear, GE Healthcare) with beta values 450 and 600 as suggested in a previous study [6]. Proprietary image analysis software (Advantage Workstation Version 4.7, GE Healthcare) was used to generate maximum intensity projection (MIP) images in anteroposterior orientation.

### Manual assessment of image noise and image quality

One reader (A.G., board-certified radiologist with 6 years of experience in diagnostic imaging) measured the pixel-wise standard deviation of a semi-automated cubicle VOIs (2 × 2 × 2 cm^3^) in the right liver lobe and in lung parenchyma, avoiding focal lesions and vasculature. Two readers (M.M. and S.S., board-certified radiologists and/or nuclear medicine physicians with 9 and 6 years of experience in diagnostic imaging respectively) reviewed all MIP images per patient in consensus. Each image was assigned the label “sufficient image quality” if both readers rated the image quality sufficient, and the label “insufficient image quality” if at least one reader rated the image quality insufficient. Readers were blinded to image reconstruction settings during the readout.

### Automated measurement of image noise

**Fig. 1:**
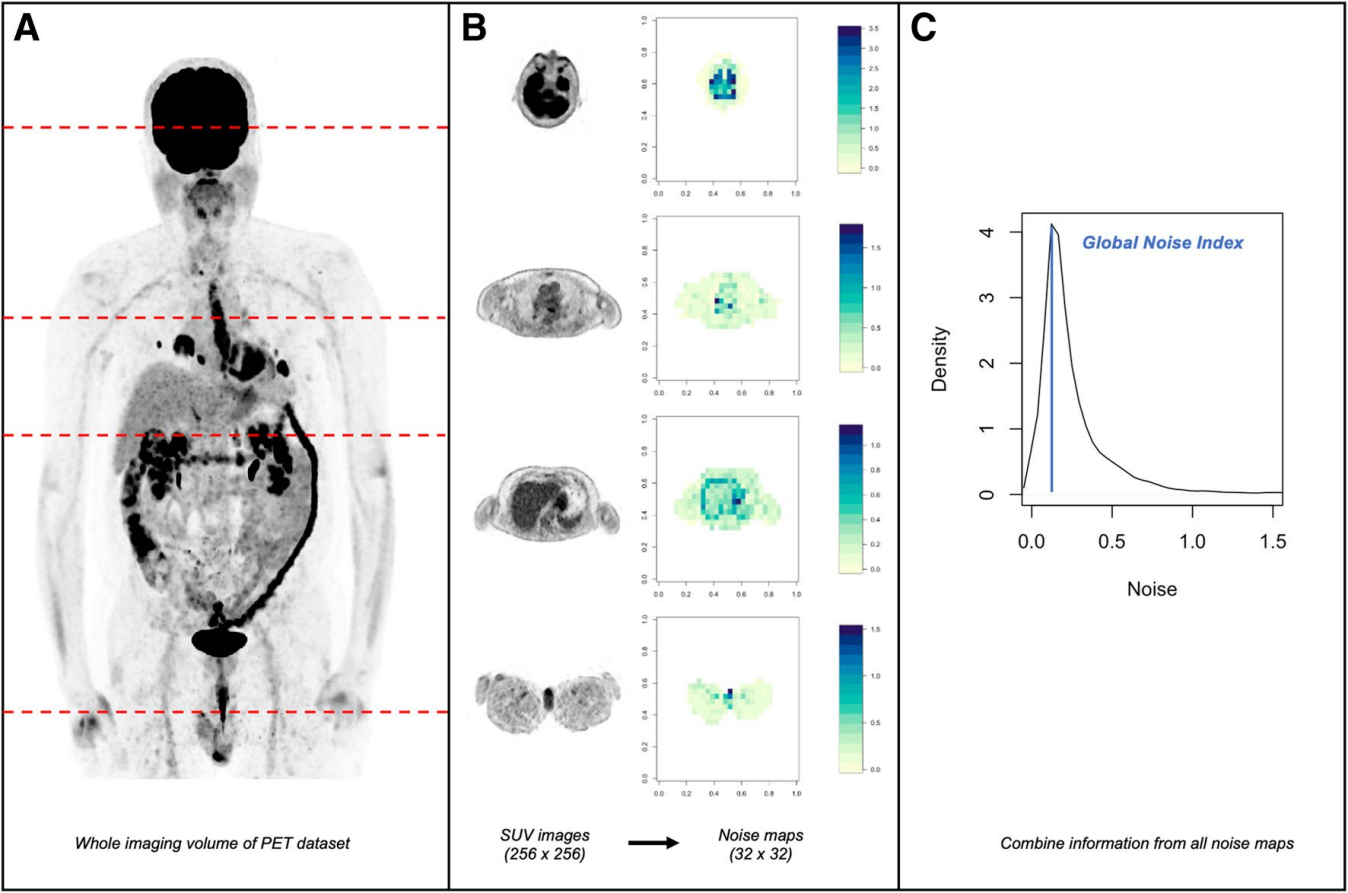
Illustration of the generation of the Globals Noise Index (GNI). **A** shows a maximum intensity projection image. To calculate the GNI, the whole imaging volume is subjected to further processing. **B** shows representative transversal image slices of the imaging volume at 4 different locations and the corresponding noise maps. **C** shows the distribution of noise values across the whole imaging volume. Specifically, the histogram is generated by considering all noise values from each image slice. The mode value of the histogram corresponds to the GNI, a single global surrogate parameter of image noise across the whole imaging volume of a given imaging dataset

An automated algorithm previously used for global image noise measurements in clinical CT examinations [12–14] was adapted for the analysis of SUV images from [^18^F]FDG PET. This algorithm builds on an approach originally described by Christianson et al [15] and was recently implemented in the open-source statistics programming language R (version 4.1.0, R Foundation for Statistical Computing) [16]. On both CT images and SUV images of PET, the standard deviation of pixel/voxel values in a given region is declared as noise [6, 12, 13]. Thus, the exact approach of the original algorithm designed for CT imaging may also be used for SUV images of PET. A visual representation of the method is provided in Fig. 1. In brief, the original SUV images from [^18^F]FDG PET are first subjected to a thresholding procedure, in which all voxels that are not part of patient tissue are excluded (part A of Fig. 1). This procedure is performed slice-by-slice as the algorithm loops through all images of a dataset (part B, left side of Fig. 1). Then, to generate so-called noise maps, the SUV images are resampled slice-by-slice to a lower matrix size so that a novel pixel (so-called macro pixel) in a resampled image (i.e., noise map) contains information from 64 pixels (i.e., 8 × 8 pixels) of the original SUV image. Importantly however, the value assigned to each of these novel macro pixels in the noise maps corresponds to the standard deviation of the SUV values of the 64 pixels contained in the original SUV image. Thus, each noise map contains locally resolved standard deviation values of the original SUV images which should provide an accurate representation of the image noise (part B, right side of Fig. 1).

From these noise maps (i.e., one noise map per slice), a histogram of the noise distribution across the whole patient is computed. Importantly, all noise values from each noise map of each slice are considered for the computation of the histogram (part C of Fig. 1). From this histogram (typically showing a right-skewed distribution), the mode value is extracted representing the global image noise level (so-called Global Noise Index, GNI). Notably, the histogram is right-skewed because noise sharply increases at anatomical borders (for example, if the standard deviation of SUV values is computed across a bordering area such as thoracic wall and lung tissue). Consequently, these few pixels in the noise maps covering anatomical borders will have very high noise values. However, because most pixels cover homogeneous tissue in which noise should be relatively lower, the histogram rises sharply at lower noise values. Thus, by using the mode value, the noise distribution in homogeneous tissue is accurately and effectively represented by the GNI (part C of Fig. 1).

**Fig. 2:**
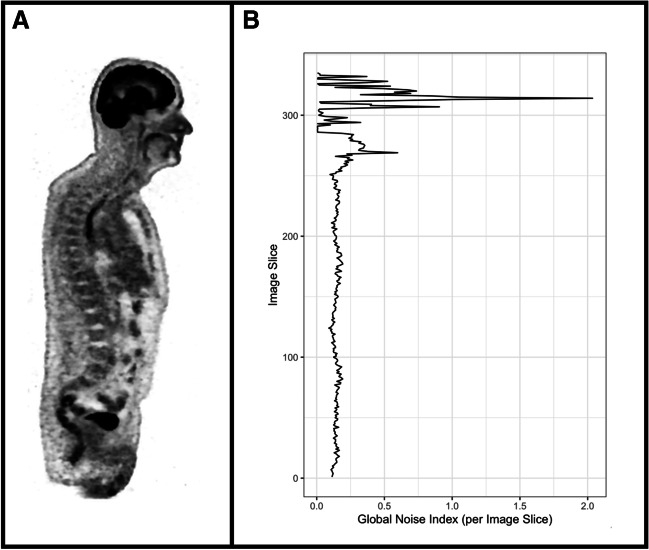
Illustration of the Global Noise Index (GNI) as computed slice-wise. **A** shows a coronal image slice. **B** shows the GNI as calculated speerately for each image slice. While not considered for this current study, a slice-wise computation of the GNI would allow the user to analyze specific anatomical regions in terms of their image noise level

Notably, for the GNI as computed in the current study, all noise values from each noise map of each image slice are considered. However, theoretically, the GNI could also be computed from individual image slices (Fig. 2). While not investigated in our study, this would allow the user to focus on the noise levels of individual anatomical regions.

### Statistical analysis

All statistical analyses were performed in the open-source statistics programming language R (version 4.1.0, R Foundation for Statistical Computing) [16]. Categorical variables are expressed as frequency distribution. Continuous variables are presented as mean ± standard deviation. As absolute values between GNI and manual noise measurements may differ, we quantified whether the distribution of noise values was similar between GNI and manual measurements irrespective of the absolute values. To this extent, noise values from GNI and manual measurements were first standardized (*z*-scoring). Then after standardization, the two-sample Kolmogorov–Smirnov tests modified for paired data were computed to compare the distribution of noise values between GNI and manual measurements of the liver and lung.

Furthermore, to further benchmark the GNI relative to manual measurements, we quantified the correlation between GNI and manual measurements in liver and lung parenchyma (i.e., without prior standardization of noise values) by computing Spearman’s rank correlation coefficients. Coefficients were interpreted according to Chan [17, 18] as follows: at least 0.8 very strong, 0.6 up to 0.8 moderately strong, 0.3 to 0.5 fair, less than 0.3 poor. To assess whether the GNI can differentiate between sufficient and insufficient image quality as determined subjectively by expert readers, receiver operating characteristic analysis was performed. The area under the curve (AUC) was computed and sensitivity and specificity were calculated at a cutoff value maximizing Youden’s index. Two-sided *p*-values of < 0.05 were considered statistically significant.

## Results

### Study cohort

Thirty-eight patients were included in our retrospective study. The mean injected [^18^F]FDG-activity was 249.3 ± 57.8 MBq, and images were acquired 58.7 ± 8 min after injection. The mean body mass index was 26 ± 5.5 kg/m^2^ (range: 15–37 kg/m^2^). Demographic data of the cohort are summarized in Table 1. Of the overall 380 imaging datasets, 268 were rated “sufficient image quality” and 112 were rated “insufficient image quality.”

**Table 1:** Demographic data of study subjects (*n* = 38)

Female/male, *n* (%)	13 (34.2%)/25 (65.8%)
Age, years	63 ± 14
Body weight, kg	79.2 ± 19
Body height, m	1.74 ± 0.1
BMI, kg/m^2^	26 ± 5.5
Blood glucose level at time of injection, mg/dl	102 ± 19
Injected F18-FDG dose, MBq	249.3 ± 57.8
Scan time post injection, min	58.7 ± 8

Values are given as absolute numbers and percentages in parenthesis or mean ± standard deviation

*BMI*, body mass index; *MBq*, megabecquerel

### Correlation of GNI and manual noise measurements

**Fig. 3:**
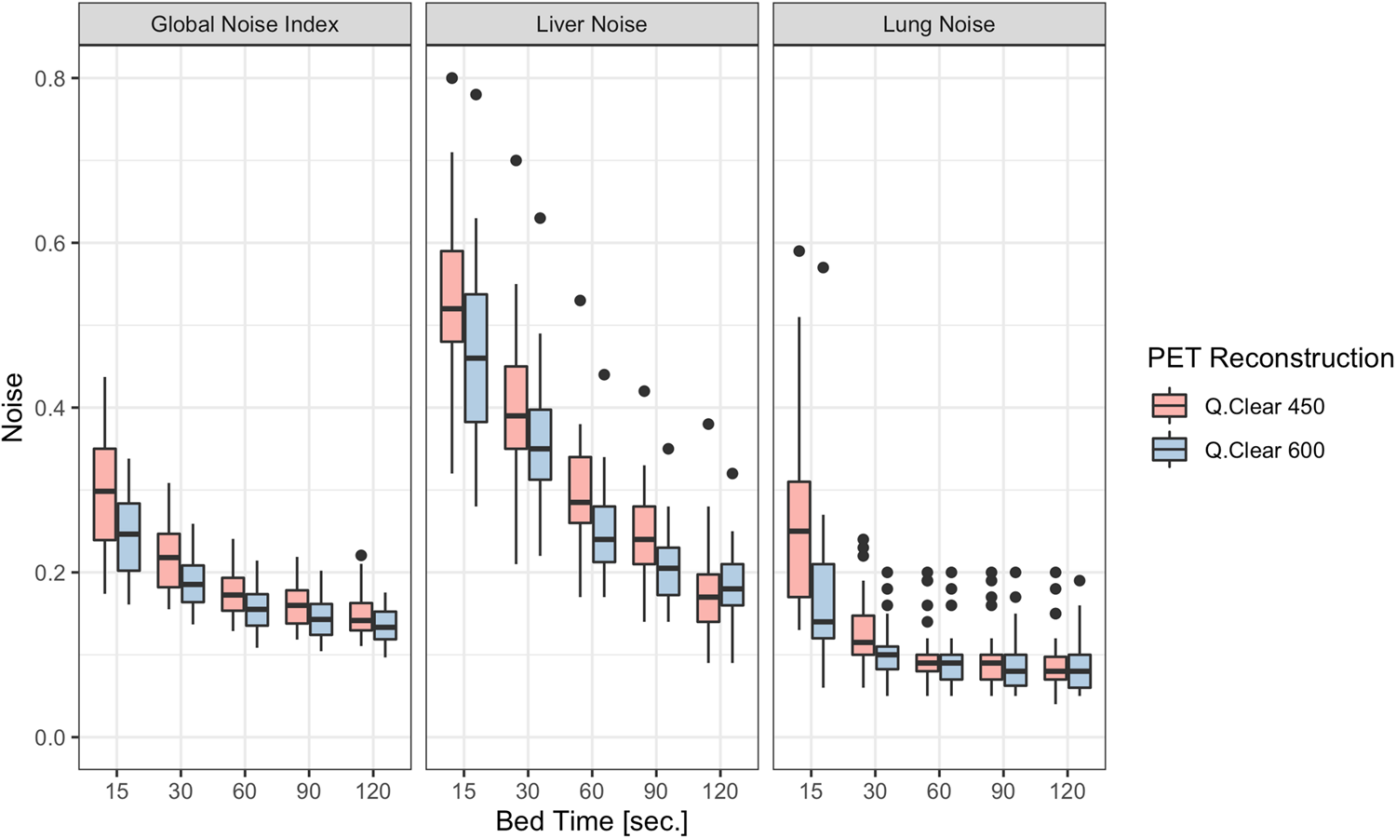
Bar plots illustrating noise distribution as a function of bed time separately for GNI and manual noise measurements in liver and lung parenchyma. The different reconstruction kernels are shown in red (Q.Clear 450) and green (Q.Clear 600)

An overview of the GNI and manual noise measurements in liver and lung parenchyma by reconstruction and bed time is given in Table 2 and illustrated in Fig. 3. The behavior of GNI values and noise values from manual measurements was as expected. Noise values decreased consistently from Q.Clear 450 to Q.Clear 600, irrespective of bed time, and noise values decreased consistently with increasing bed time, irrespective of reconstruction type (Q.Clear 450 vs Q.Clear 600).

**Table 2:** Overview of Global Noise Index and manual noise measurements by bed time and reconstruction

Characteristic	Bed time 15 s		Bed time 30 s		Bed time 60 s		Bed time 90 s		Bed time 120 s	
	Q.Clear 450	Q.Clear 600	Q.Clear 450	Q.Clear 600	Q.Clear 450	Q.Clear 600	Q.Clear 450	Q.Clear 600	Q.Clear 450	Q.Clear 600
Global Noise Index	0.30 ± 0.07	0.24 ± 0.05	0.22 ± 0.04	0.19 ± 0.03	0.18 ± 0.03	0.16 ± 0.03	0.16 ± 0.03	0.14 ± 0.02	0.15 ± 0.03	0.14 ± 0.02
Manual image noise measurement (liver)	0.58 ± 0.20	0.46 ± 0.10	0.40 ± 0.09	0.35 ± 0.08	0.30 ± 0.06	0.25 ± 0.05	0.24 ± 0.05	0.21 ± 0.04	0.18 ± 0.06	0.18 ± 0.04
Manual image noise measurement (lung)	0.28 ± 0.16	0.17 ± 0.09	0.13 ± 0.04	0.10 ± 0.03	0.10 ± 0.03	0.09 ± 0.03	0.09 ± 0.03	0.09 ± 0.03	0.09 ± 0.03	0.08 ± 0.03

Values are means ± standard deviations

*Q.Clear*, block sequential regularized expectation maximization PET reconstruction algorithm

In terms of the similarity of the distribution of noise values (irrespective of the absolute noise values, i.e., after the standardization procedure), there was no significant difference between the GNI and manual liver measurements (*p* = 0.51 for Q.Clear 600 and *p* = 0.84 for Q.Clear 450). However, the distribution of values differed significantly, both between manual liver and lung measurements (*p* = 0.03 for Q.Clear 600 and *p* < 0.001 for Q.Clear 450) and between GNI and manual lung measurements (*p* = 0.09 for Q.Clear 600 and *p* < 0.001 for Q.Clear 450). This implies that although the absolute noise values may differ (considerably), the GNI closely resembles the behavior of noise values derived from manual liver measurements. Specifically, GNI showed a fair to moderately strong correlation with manual noise measurements in liver parenchyma (*r* = 0.6 in Q.Clear 450, *r* = 0.54 in Q.Clear 600, all *p* < 0.001), and a fair correlation with manual noise measurements in lung parenchyma (*r* = 0.52 in Q.Clear 450, *r* = 0.33 in Q.Clear 600, all *p* < 0.001).

### Classification performance of GNI for image quality

**Fig. 4:**
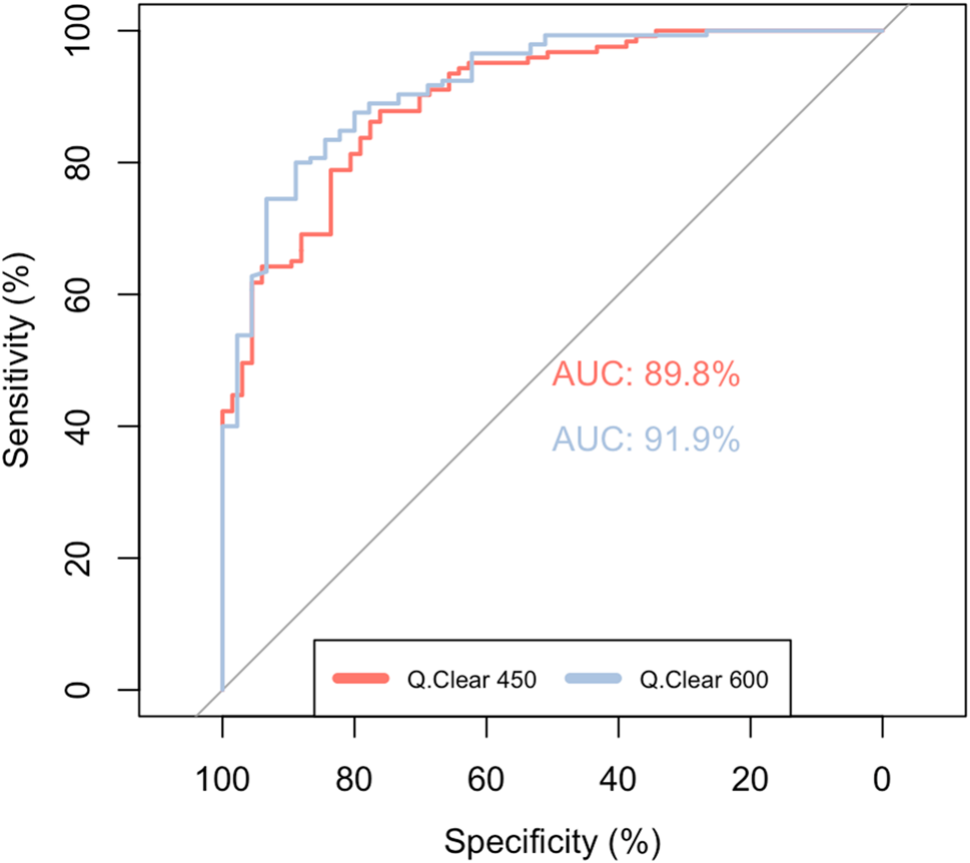
Receiver operating characteristic (ROC) curves for the classification performance of GNI for image quality. Curves are shown for both Q.Clear 450 and Q.Clear 600

The AUC of the GNI for the classification of subjective image quality using reader-based assessment as target was 0.898 (95% confidence interval (CI): 0.855–0.942) in Q.Clear 450 and 0.919 (CI: 0.875–0.962) in Q.Clear 600 (Fig. 4). Maximizing the Youden index, the sensitivity and specificity for the GNI were 88% and 76% for Q.Clear 450 images, using a cutoff value of 0.21, and 80% and 89% for Q.Clear 600 images, using a cutoff value of 0.18.

## Discussion

In this study, we aimed to develop and assess the performance of an algorithm that enables the automated computational estimation of the noise level across the whole imaging volume of PET datasets.

The major findings of our study are as follows: First, an algorithm delivering a measure of noise on the whole imaging volume that builds on an approach originally developed for clinical CT imaging by Christianson et al [15] can successfully be adapted for clinical PET imaging. Importantly, computationally derived noise values closely resemble the behavior observed from manual measurements in the liver and are correlated with manual measurements in liver and lung parenchyma in terms of absolute values. Second, the performance of the algorithm for classification of image quality compared to subjective reader’s evaluation was very good.

Generally in PET, image noise, among other factors, is considerably affected by administered activity and acquisition time. Recent studies suggest that by implementing latest generation hardware and software for PET imaging and by using BMI-based dosage protocols, acquisition time, and administered tracer dose can be continuously decreased without compromising image quality [19–23]. In this regard, it is essential to closely monitor and benchmark new protocols against the current clinical standard in terms of image quality in order to ensure that diagnostic image quality is preserved in clinical routine. For example, this is very important when testing new image reconstruction algorithms or their parameters. Here, we used beta values of 450 and 600 for our reconstruction algorithm based on previous recommendations [6]. With higher beta values, for example, the noise would decrease further, which could then theoretically be quantified with our approach.

While image quality depends on various factors, including subjective preferences, noise is an important surrogate parameter that is commonly used as a reliable and objective metric to assess the quality of PET images [7, 8]. Specifically, noise is not only an important marker of image quality itself, but also plays a crucial role in calculating quantitative contrast measurements as important surrogate markers of image quality, such as the signal-to-noise ratio or contrast-to-noise ratio [6, 8, 24].

Thus, a large-scale evaluation of image noise is highly desirable as an important marker of image quality, ideally in a high-throughput and non-reader-dependent fashion. The approach presented in our study may be a promising candidate for this task. As our method enables an accurate and fully automated estimation of the global noise level across the whole imaging volume, PET imaging data can be benchmarked, evaluated, and compared both longitudinally and between different scanners and vendors. This may be especially useful for quality standardization and protocol harmonization across different institutions.

Importantly, our algorithm potentially allows the user to compute the noise level of individual anatomical regions or the noise level of single bed positions. This may be of interest as our metric can then be compared with the detectability and visualization of individual organ-wise pathologies or, in the latter case, can be used to optimize the acquisition of remaining bed positions based on the noise level of the first bed position. Additionally, it should be noted that our approach could also be valuable for the assessment of images acquired as part of dynamic PET imaging, since the noise level of individual slices can also be assessed as shown exemplarily in Fig. 2.

Our study has some limitations. Its retrospective nature and single-center scale, the relatively small cohort, the unbalanced dataset, and the fact that only one scanner from a single vendor was included all limit generalizability. Further studies are necessary to validate the algorithm, especially across different scanners. In this regard, a correlation with more advanced metrics of image quality, such as the noise-equivalent count rate (NECR) [25, 26], is of great interest. Second, manual noise measurements were only performed by a single reader using a specific measurement procedure. We acknowledge that our results may have been impacted by the choice of measurement procedure (i.e., specific type of reader, choice of VOI size, etc.). Last, image quality was subjectively assessed by only two readers in our study and may differ among a wider range of interpreters.

## Conclusion

An automated computational noise measurement algorithm provides an accurate and meaningful estimation of the global noise level encountered in clinical PET imaging datasets. The algorithm provides noise values that correlate with manual VOI-based noise measurements and provides high performance for the determination of subjective image quality.

## Abbreviations

BSREM: Block sequential regularized expectation maximization reconstruction
CI: 95% Confidence interval
F18-FDG PET: ^18^F-Fluorodeoxyglucose positron emission tomography
GNI: Global Noise Index
Q.Clear: Trade name of block sequential regularized expectation maximization reconstruction
SUV: Standardized uptake values

## Declarations

### Guarantor

The scientific guarantor of this publication is PD Dr. Michael Messerli.

### Statistics and biometry

One of the authors has significant statistical expertise.

### Study subjects or cohorts overlap

Some study subjects or cohorts have been previously reported in “Automated F18-FDG PET/CT image quality assessment using deep neural networks on a latest 6-ring digital detector system” (Scientific reports, https://doi.org/10.1038/s41598-023-37182-1).

### Methodology

• retrospective

• experimental

• performed at one institution
